# Targeting the innate repair receptor to treat neuropathy

**DOI:** 10.1097/PR9.0000000000000566

**Published:** 2016-08-09

**Authors:** Albert Dahan, Michael Brines, Marieke Niesters, Anthony Cerami, Monique van Velzen

**Affiliations:** a Department of Anesthesiology, Leiden University Medical Center, Leiden, the Netherlands; b Araim Pharmaceuticals, Inc, Tarrytown, NY, USA

**Keywords:** Neuropathic pain, Neurogenic inflammation, Small fiber neuropathy, EPO, CD131, ARA290, Sarcoidosis, Diabetes mellitus

## Abstract

The innate repair receptor (IRR) is a heteromer of the erythropoietin receptor and the β-common (CD131) receptor, which simultaneously activates anti-inflammatory and tissue repair pathways. Experimental data suggest that after peripheral nerve injury, the IRR is upregulated in the spinal cord and modulates the neurogenic inflammatory response. The recently introduced selective IRR agonist ARA290 is an 11-amino acid peptide initially tested in animal models of neuropathy. After sciatic nerve injury, ARA290 produced a rapid and long-term relief of mechanical and cold allodynia in normal mice, but not in animals with a β-common receptor knockout phenotype. In humans, ARA290 has been evaluated in patients with small fiber neuropathy associated with sarcoidosis or type 2 diabetes (T2D) mellitus. In patients with sarcoidosis, ARA290 significantly improved neuropathic and autonomic symptoms, as well as quality of life as assessed by the small fiber neuropathy screening list questionnaire. In addition, ARA290 treatment for 28 days initiated a regrowth of small nerve fibers in the cornea, but not in the epidermis. In patients with T2D, the results were similar to those observed in patients with sarcoidosis along with an improved metabolic profile. In both populations, ARA290 lacked significant adverse effects. These experimental and clinical studies show that ARA290 effectively reprograms a proinflammatory, tissue-damaging milieu into one of healing and tissue repair. Further clinical trials with long-term treatment and follow-up are needed to assess the full potential of IRR activation by ARA290 as a disease-modifying therapy in neuropathy of various etiologies.

## 1. Introduction

Although our knowledge of the pathophysiology of neuropathic pain, ie, pain caused by a lesion of the somatosensory nervous system, has increased significantly over the last decades, it remains difficult to control the chronic pain condition, which affects many patients. Neuropathic pain is observed in a large variety of disorders that vary from metabolic and infectious disorders, which affect the small, afferent sensory nerve fibers (eg, diabetes mellitus, sarcoidosis, leprosy), to diseases of the central nervous system (eg, stroke, multiple sclerosis, traumatic brain, or spinal cord injuries). Patients with neuropathic pain share a spectrum of clinical symptoms, irrespective of the underlying disease, including spontaneous (burning, electrical) pain, allodynia (increased sensitivity to nonnoxious stimuli such as intolerance of bed sheets), and hyperalgesia (increased sensitivity to noxious stimuli). In addition, patients with neuropathic pain often display symptoms of anxiety, depression, sleep disturbance, and social isolation, all further reducing their quality of life. Although multiple therapeutic options are available, only a minority of patients (30%-50%) achieve moderate to adequate pain relief with an average reduction in pain score of 30% to 50%.^17,21,24,25^ Therapies include pharmacotherapy (anticonvulsants, antidepressants, opioids, topical agents such as capsaicin and lidocaine), interventional therapies (eg, spinal cord stimulation), as well as, physiotherapy, and cognitive interventions, with patients often exposed to multiple consecutive treatments.^2^ Notably, no disease modification treatment, ie, leading to nerve repair, have been developed. Furthermore, current pharmacotherapies come with severe side effects that reduce patient compliance, which consequently is an additional cause of failed therapy.

## 2. Neurogenic inflammation

**Figure 1.**
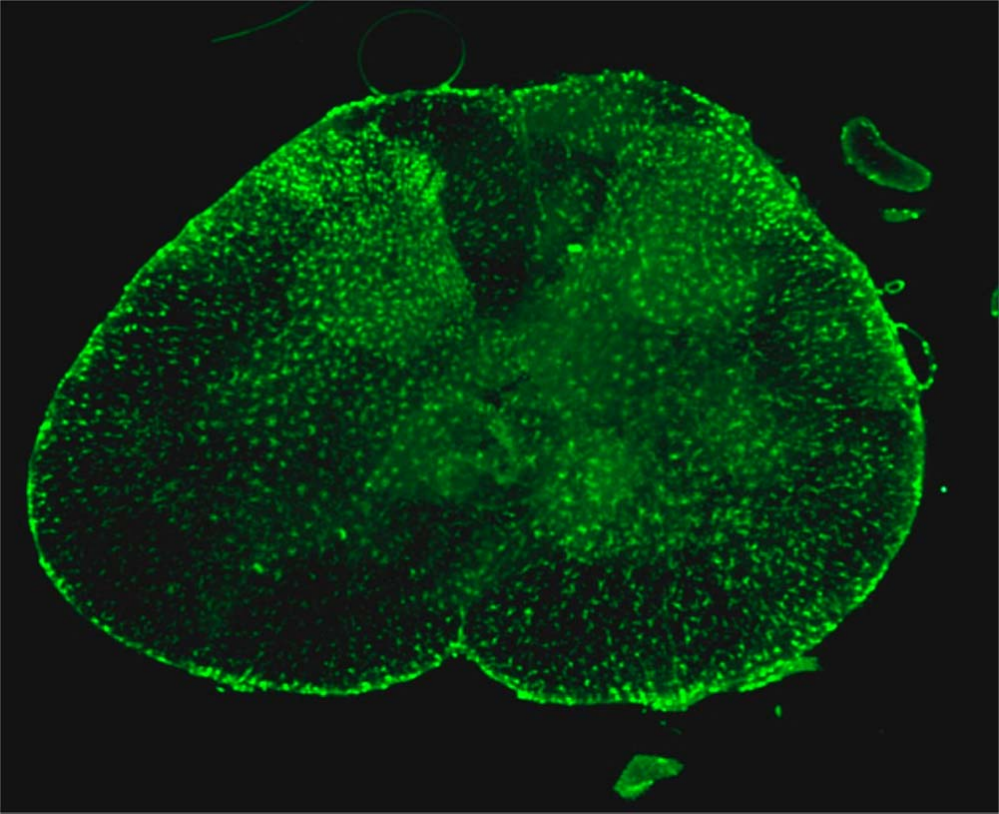
Iba-1 immunoreactivity (green) in the L5 spinal cord segment of a rat 2 weeks after spared nerve injury of the left hind limb. Activated microglial cells in the dorsal horn and corresponding motor pool are evident on the affected side. (From Ref. 51; doi: 10.1186/1744-8069-10-13).

An important feature of neuropathic pain is the occurrence of peripheral and/or central inflammation.^1,22,37,53^ Inflammation is thought to be caused by nerve damage and to maintain resulting painful symptoms, eventually resulting in peripheral and/or central sensitization with persistent allodynia and hyperalgesia. For example, there is ample evidence from animal studies that peripheral nerve injury triggers a spinal inflammatory response with the release from nociceptive spinal neurons of proinflammatory molecules, such as fractalkine, chemokine (C-C motif) ligand-2 (CCL2), and tumor necrosis factor (TNF-α), which results in the invasion of resident macrophages (microglial cells) and monocytes from peripheral blood toward the spinal cord level of the peripheral nerve lesion site.^26,39,46,58^ Consequently, at that location, microglial cells increase in number and along with astrocytes show signs of heightened reactivity (Fig. 1). In addition, members of the transient receptor potential channel family, especially transient receptor potential vanilloid 1 (TRPV1), play a critical role in initiating and maintaining neuroinflammation.^36^ Although the release of proinflammatory mediators from nociceptive neurons initiates a glial response, the activated microglia subsequently maintain and amplify the inflammatory response by the release of chemokines and cytokines (eg, interleukins 1β and 6, TNF-α).^19,20,40,48^ Importantly, the inflammatory response is not restricted to the level of the nerve injury but also moves in a cranial and caudal direction with concomitant symptoms of spreading mechanical allodynia.^3,51,59^ In addition, there is evidence of contralateral dissemination of the inflammatory response.^41,44^ Although the neuroinflammatory response may eventually subside, neuroplastic changes occur in pain sensory and modulatory networks which cause the persistence of symptoms. These findings of neurogenic inflammation are mirrored by one case report in a deceased patient who had suffered from long-standing complex regional pain syndrome.^18^ His autopsy revealed heightened spinal cord glial activity. The body of evidence that is described above strongly suggests that inhibition of the activated inflammatory cells in the spinal cord may be an important mechanism to relieve neuropathic symptoms. Evidence for this is found in the observation that drugs, which stabilize activated microglial cells, such as ketamine, local anesthetics, or minocycline, reduce mechanical allodynia after experimental damage to large peripheral nerves.^32,50,56,61^ The effect of ketamine on neuropathic pain has previously been ascribed to its exclusive effect at the N-methyl-d-aspartate (NMDA) receptor. However, we recently showed that ketamine reduces the production of inflammatory molecules within the spinal cord after sciatic nerve injury with a concomitant decrease in mechanical allodynia.^49^ These effects were unrelated to NMDA receptor antagonism (see Section 5, penultimate paragraph).

## 3. The innate inflammatory response

**Figure 2.**
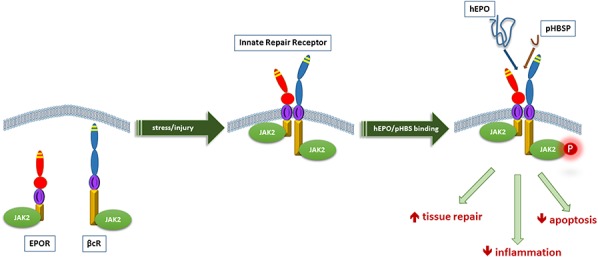
Biology of the innate repair receptor is characterized by temporal and spatial separation of the receptor subunits, which associate to form the innate repair receptor and the endogenous ligand, hyposialated erythropoietin (hEPO). After tissue stress or injury, receptor subunits located intracellularly are rapidly recruited to the cell surface and spontaneously associate. After occupancy by an activating ligand, eg, ARA290 (pyroglutamate helix B surface peptide [pHBSP]), tissue protection and repair are simultaneously activated. (From Ref. 14; doi: 10.1016/j.pharmthera.2015.02.005).

Tissue injury, such as caused by pathogen invasion, ischemia, traumatic injury, or peripheral nerve damage, activates an innate immune response with attraction of inflammatory cells (macrophages) and the subsequent release of proinflammatory cytokines (eg, TNF-α), which are aimed at the isolation and destruction of toxins, pathogens, and damaged tissue.^6,13^ This process involves the release of reactive oxygen species, vascular thrombosis, and edema formation and the mobilization of additional inflammatory cells. To balance this destructive process, dampen the inflammatory process, and counteract collateral damage (apoptosis of nearby nonaffected cells), anti-inflammatory molecules are upregulated in response to the tissue injury, one of the most important ones being the cytokine erythropoietin (EPO).^5,6^ Note that the local synthesis and release of EPO after tissue injury is distinct from its endocrine effect on the hematopoietic system, which is related to the release of low concentrations of EPO by the kidney. Furthermore, locally produced EPO is chemically distinct from renal EPO, because of a reduced degree of sialylation, which acts to significantly reduce its half-life. Erythropoietin acts through the erythropoietin homodimer receptor (EPOR_2_) on erythrocyte precursor cells in the bone marrow with high affinity to modulate the production of mature red blood cells; activation of the EPOR_2_ inhibits apoptosis of red cell precursor cells. In contrast, the paracrine/autocrine EPO-induced tissue-protective effects are mediated through activation of the innate repair receptor (IRR), which is a member of the Type 1 cytokine receptor family.^5,6^ The IRR is a heteromeric receptor that comprises β-common (CD131) and EPO receptor subunits (reviewed in Ref. 14). The IRR is typically not expressed by normal tissues, but rather is locally upregulated in response to tissue injury, hypoxia, or metabolic stress. Because the affinity of EPO for the receptor is low, high local concentrations of EPO are required at the site of injury. Activation of the IRR simultaneously actives anti-inflammatory and tissue repair pathways (Fig. 2).

A number of studies confirmed the tissue-protective role of exogenously administered EPO in experimental models of renal, cardiac, retinal, and neural damage (reviewed in Refs. 5,6,14). For example, motor function after spinal cord compression in mice responded with a full recovery after a single dose of carbamylated EPO, which because of its chemical modification selectively activates only the IRR.^8^ Recovery was absent in homozygous βCR knockout mice (βCR^−/−^), ie, mice lacking the IRR, indicative of the molecular mechanism of action of EPO. Exogenous EPO is also effective in animal models of painful neuropathy with a rapid recovery of sensory functions and reduction of allodynia.^4,11,12,23,34,35^ However, clinical studies indicate that the use of recombinant EPO is associated with serious side effects, such as arterial and venous thrombosis, hypertension, stroke, and myocardial infarction.^15^ Hence, the use of exogenous EPO has not been pursued seriously in clinical studies requiring extended dosing.

## 4. Nonhematopoietic analogues of EPO: ARA290

To overcome the hematopoietic and thrombotic effects of EPO, several nonhematopoietic IRR-activating analogues have been developed such as carbamylated EPO, and ARA290, also known as pyroglutamate helix B surface peptide.^9,43^ These compounds do not bind to the EPOR_2_ but selectively activate the IRR and consequently possess potent tissue-protective properties.^9,38^ ARA290 is a molecule that has been evaluated extensively in neuropathic pain.^43,57^ ARA290 (Araim Pharmaceuticals, Inc, Tarrytown, NY) is an 11-amino acid linear peptide with a molecular weight of 1257 Da (Pyr-Glu-Glu-Leu-Glu-Arg-Ala-Leu-Asn-Ser-Ser-OH) that was designed to separate EPO's hematopoietic and thrombotic effects from its anti-inflammatory effects. ARA290 mimics the configuration in space of the B-helix of the EPO molecule, which interacts with the IRR.^9^ The pharmacokinetics of ARA290 in humans shows an elimination half-life of ∼20 minutes after subcutaneous administration and just 2 minutes after an intravenous injection.^27,43^ Similar observations were made in animal studies and suggest that ARA290 rapidly reaches its target site and activates a master switch (the IRR), which subsequently initiates a cascade of events ultimately resulting in clinical effects.^9,14,27^ ARA290 may activate IRRs within the central nervous system (animal studies confirm the rapid transfer of ARA290 across the blood–brain barrier), as well as at peripheral sites.

Cooperation between Anthony Cerami, Michael Brines, and the Experimental Pain Research Group of the Department of Anesthesiology at Leiden University Medical Center has led to a series of experimental and clinical studies on ARA290's effect in neuropathy.

## 5. Targeting the innate repair receptor in neuropathic pain: animal studies

Erythropoietin improves neuropathic pain symptoms in a variety of experimental neuropathic pain models. Recombinant human EPO (*rh*EPO) improved sensory nerve function in streptozotocin-induced diabetic neuropathy.^4^ Erythropoietin reduced allodynia and improved motor function in rodents after L5 spinal nerve transection, dorsal root ganglion crush injury, and chronic constriction injury.^12,23,34^ Importantly, signs of spinal inflammation were reduced by systemic administration of *rh*EPO resulting in less reactive glial cells and decreased release of TNF-α, interleukin-1β, and NF-κB activation in the spinal cord and at supraspinal sites. Also, peripheral effects are observed after *rh*Epo with the reduction of intraepidermal nerve loss in diabetic neuropathy and reduced Schwann cell TNF-α expression in chronic constriction injury.^4,11^ In contrast, in a rat neuritis model, ARA290 prevented the development of mechanical allodynia but did not reduce TNF-α mRNA production in the affected nerve, suggestive of a central effect of ARA290.^45^ In addition, ARA290 has been shown to promote repair of small autonomic nerve fibers within sympathetic ganglia in a mouse model of diabetic neuropathy.^47^

**Figure 3.**
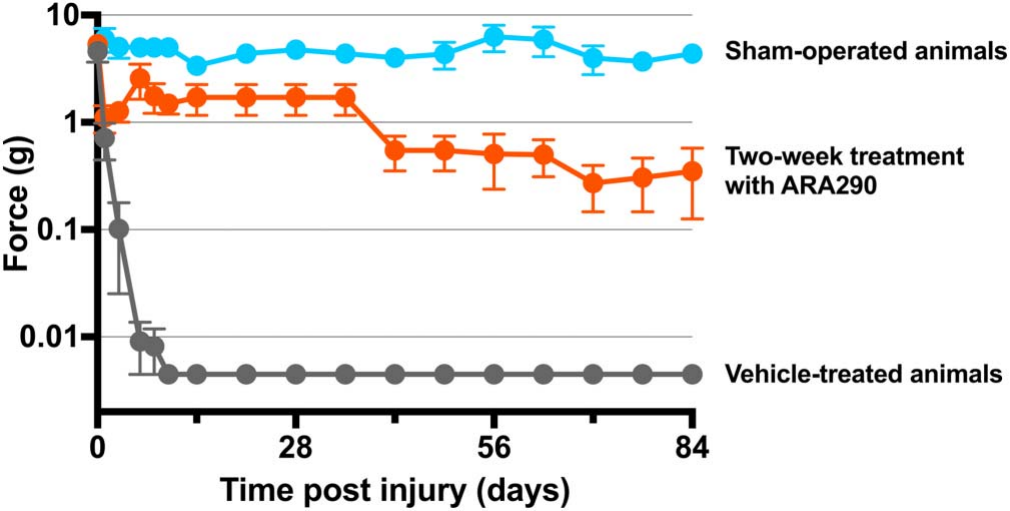
Innate repair receptor activation reduces mechanical allodynia after spared nerve injury (SNI) in the rat. Animals were sham operated (blue) or received SNI and vehicle (grey) or SNI and 60 µg/kg ARA290 (red), both administered on days 1, 3, 6, 8, and 10 postsurgery (*P* < 0.0001 compared with vehicle). (Redrawn from Ref. 51; doi: 10.1186/1744-8069-10-13).

**Figure 4.**
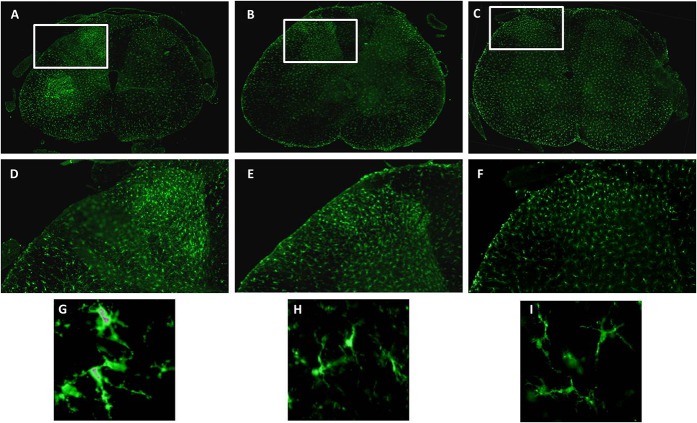
Effect of targeting the innate repair receptor on Iba-1 immunoreactivity in the L5 spinal cord segment of animals 2 weeks after spared nerve injury (SNI). Microscope image of Iba-1 immunoreactivity in the L5 spinal cord segment of rats 2 weeks after SNI. (A-C) Low-power magnifications, (D-F) detailed images of (A-C) as indicated by the white rectangles, and (G-I) high-power magnifications of the spinal cord of animals that underwent SNI (A, D and G). SNI and vehicle treatment, B, E, and H. SNI and treatment with 30 μg/kg ARA290, C, F, and I. Sham surgery without treatment. The left-hand parts of the images represent the side of the injured sciatic nerve. (From Ref. 51; doi: 10.1186/1744-8069-10-13).

We tested ARA290 in the spared nerve injury (SNI) model in mice and rats.^49–51^ In this model, 2 of the 3 branches of the sciatic nerve are cut, the tibial and common peroneal branches, while one, the sural branch, is left intact. This model produces a rapid development of mechanical and cold allodynia in the affected limb, which persists for at least 4 months. Weekly intraperitoneal injections with ARA290 produced effective and long-lasting relief of both forms of allodynia.^49^ Also, after just 2 weeks of intensive ARA290 treatment, long-term analgesic effects were observed (Figs. 3 and 4). However, over time (>6 weeks after nerve injury), a slow return of allodynia was observed. In βCR^−/−^ mice, ARA290 was without effect on allodynia, indicative that the site of action of ARA290 is the IRR.^49^ In a next set of experiments,^51^ the effect of ARA290 on the SNI-induced spinal inflammatory response was studied. In week 2 to 20 after nerve injury, a spreading microglial response was observed from L5 to L2-L6, which was effectively suppressed by ARA290. Finally, we showed that upregulation of spinal inflammatory mediator CCL2, and markers Iba-1 and GFAP and NMDA receptor subunits (NR1, NR2A, and NR2B) due to nerve injury was attenuated by ARA290 (all measurements made were based on gene expression),^50^ suggestive of an effect of ARA290 on the inflammatory response which involved both microglia and astrocytes.

We additionally tested the effect of subanesthetic concentrations of ketamine in our SNI model and observed that ketamine reduced mechanical allodynia as well as neuropathy-related inflammatory markers and NMDA receptor subunits in a similar manner compared with ARA290.^50^ Additional experiments in mice lacking the IRR showed that ketamine-induced reduced behavioral responses to acute noxious stimuli (ie, antinociception) were retained; however, ketamine was without effect on SNI-induced allodynia. We relate the antinociceptive effects of ketamine to its actions on NMDA receptor antagonism and consequently inhibition of glutamate-dependent pain signaling and possibly also to its effects on mu-opioid receptors. These same NMDA receptors seem to be unimportant for the effects of ketamine in SNI neuropathy. Like ARA290, ketamine is a potent anti-inflammatory agent and has been shown to reduce TNF-α in a murine laparoscopic model.^52^ Whether ketamine directly interacts with the IRR remains unknown at present, but it is clear that ketamine analgesia in SNI-induced chronic allodynia is dependent on a pathway, which involves the IRR.

Recently, it has been shown that ARA290 inhibits capsaicin-evoked TRPV1 channel activity in dorsal root and trigeminal ganglion cells and relieves capsaicin-induced mechanical allodynia in mice.^60^ This ion channel is a key modulator of neuroinflammation and nociception, and therefore, the beneficial effects of IRR activation likely depends at least in part on dampening TRPV1 activity of small intraepidermal nerve endings and on central effects.

## 6. ARA290 effects in small fiber neuropathy

The administration of ARA290 to humans followed as a logical step considering the positive results of the animal experiments. Clinical studies were performed after approval from the Leiden University Medical Center Institutional Review Board (LUMC, Leiden, the Netherlands). Protocols were registered in the publicly available Netherlands Trial Register (numbers 3081, 3575, and 3858). For all clinical studies, oral written informed consent was obtained from participants before study enrollment.

Initially, an open-label proof-of-concept and safety study was performed in a small number of sarcoidosis (n = 10) and type 2 diabetes (T2D) mellitus (n = 10) patients with moderate to severe neuropathy pain (pain score of >5 on an 11-point numerical rating scale).^43^ All patients were diagnosed with small fiber neuropathy (SFN) and had pain symptoms as well as signs of autonomic nerve fiber loss. All patients received 3 intravenous injections with ARA290 over one week (2 mg was administered were given on Monday, Wednesday, and Friday). No relevant adverse events were noted. Pain relief was maximal on the day of the third injection with a 25% reduction in pain score in patients with T2D (baseline score: 7.0) and 40% in patients with sarcoidosis (baseline score: 7.1). These results without apparent significant adverse effects prompted us to perform a series of placebo-controlled double-blind trials to assess the effect of ARA290 on SFN-related symptoms in patients with sarcoidosis.

Sarcoidosis is a multiorgan inflammatory illness, which is characterized by noncaseating granulomas.^28,33^ Although any organ may be affected, in more than 90% of cases the lungs are involved. Pain in sarcoidosis is often related to SFN, which selectively affects thinly myelinated Aδ- and unmyelinated C-nerve fibers.^28^ Because these small fibers subserve both sensory and autonomous function, symptoms include neuropathic pain and autonomic dysfunction. Autonomic symptoms include orthostatic dizziness, dry eyes, facial flushing, incontinence for urine, sexual dysfunction, hypo/hyperhidrosis, and restless leg syndrome among others.

In 3 randomized controlled trials, the efficacy of ARA290 was evaluated against a placebo control. The data of the first 2 studies have been analyzed and published.^16,29^ Both studies were small proof-of-concept phase 2 trials in a total of 60 patients of which 33 patients received active treatment. All patients had a confirmed diagnosis of sarcoidosis with painful SFN. In the first study (n = 22), patients were treated for 1 month with intravenous ARA290 2 mg administered 3 times weekly, with 3-month follow-up.^29^ Main endpoints were (1) pain intensity scores from the Brief Pain Inventory (BPI) questionnaire; (2) the score on the Small Fiber Neuropathy Screening List (SFNSL),^30^ a validated Dutch questionnaire for assessment of SFN in sarcoidosis. The SFNSL reports on pain-related and autonomic symptoms; and (3) quality-of-life scores from the RAND-36 questionnaire. Although pain intensity scores were similarly reduced by 2.1 (placebo; baseline score: 6.2) and 2.3 points (ARA290; baseline score: 7.1) in both treatment arms, ARA290 produced greater effects in the 2 dimensions of the SFNSL at week 4 of treatment with additionally a greater responder rate (SFNSL score reduction of 1-5 points in 83 vs 60%; >15 points reduction in 42 vs 0%. Baseline scores: ARA290 = 41.0; placebo = 30.6; of a maximum of 84). Also, the quality of life improved significantly by ARA290 treatment. No safety issues were detected.

**Figure 5.**
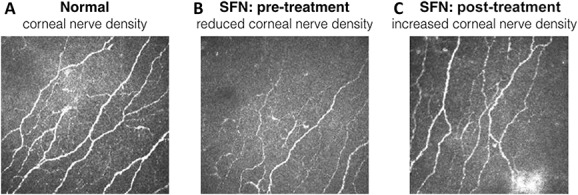
Corneal confocal microscope images obtained from a normal individual (A) compared with a sarcoidosis patient with small fiber neuropathy (SFN) before therapy with ARA290 (B) and after 4 weeks of ARA290 treatment (C). Bright linear structures correspond to fiber bundles consisting of multiple c-fibers, with the thickness of each bundle directly related to number of fibers contained within.

In the second phase 2 trial with acronym NERVARA (n = 38),^16^ subcutaneous ARA290 4 mg was administered daily for 4 weeks with a 3-month follow-up. The focus of this trial was on the effect of ARA290 on small fiber morphology as measured from skin biopsies and corneal confocal microscopy and functional capacity as assessed by the 6-min walk test. Corneal confocal microscopy is a relatively new technique used to quantify the morphology of small fibers (predominantly the unmyelinated C-fibers) in Bowman layer of the cornea.^10,31^ Corneal confocal microscopy allows determination of corneal nerve fiber area, corneal nerve fiber length (CNFL), corneal nerve fiber density (CNFD), and corneal nerve fiber branching density. The cornea may be especially sensitive to changes in the small nerve fibers of the human body as it has the highest density of small nerve fibers than all other tissues. For example, compared with the skin, the nerve fiber density is approximately 600 times higher. The corneal nerve fibers originate from the ophthalmic branch of the trigeminal nerve, and the fibers run parallel to the surface. Hence, visualization of these with a confocal microscope is relatively easy. An example of a healthy individual and patient with SFN is given in Figure 5. Corneal confocal microscopy has been applied in various forms of neuropathic pain before including T2D polyneuropathy and chemotherapy-induced polyneuropathy.^24,31,54,55^ We recently showed in patients with sarcoidosis that CNFD and CNFL are reduced compared with normal and inversely correlate with the degree to which pain interferes with daily activities (assessed by the Brief Pain Inventory questionnaire).^10^ In addition, CNFL correlated with intraepidermal nerve density of the distal (but not proximal) limb after corrections were made for age, gender, and height.

The results of the NERVARA trial indicate a significant increase in corneal nerve fiber area by 14% within the 4-week treatment period (relative to a small decrease after placebo treatment) without affecting proximal and distal limb intraepidermal nerve fiber densities.^16^ These data are important for 2 reasons. First, ARA290 initiates a rapid regrowth of small nerve fibers in the cornea, which is a sign of tissue healing and restoration as earlier observed in experimental studies. Second, the corneal nerve fibers seem to be more sensitive to regeneration than skin fibers, and consequently, the cornea is the more appropriate location to assess not only the state of small fiber pathology but also assess the effect of treatment. Moreover, the technique is simple and noninvasive and may be repeated frequently without causing damage.

Functional capacity as evaluated by the 6-minute walk test was reduced by approximately 30% in the sarcoidosis patients with SFN compared with normal age and gender-matched controls. After 28 days of ARA290 administration, 50% of patients receiving ARA290 could walk at least 25 m more, compared with only 12% in placebo-treated patients. Additional results of the NERVARA trial included an improvement of SFN symptoms as assessed by the SFNSL questionnaire with an ameliorating effect in the 2 queried domains, pain and autonomic symptoms. The results of these 2 small-randomized placebo-controlled trials are promising and indicate that the tissue-restorative peptide ARA290 produces improvement in neuropathic pain and autonomic symptoms, quality of life, and exercise capacity, and initiates the process of nerve fiber regeneration in the cornea. Larger trials are needed to further strengthen the evidence of a role of ARA290 in the long-term treatment of neuropathic pain.

The data from one additional randomized controlled trial on ARA290 have recently become available.^7^ Forty-eight T2D patients with moderate to severe SFN-induced chronic pain were treated with daily subcutaneous injections of ARA290 4 mg or placebo for 28 days. The results of the trial were similar to those obtained earlier in sarcoidosis patients with improvements in neuropathic symptoms, an increase in CNFD in patients with an initial reduction in CNFD. Furthermore, an improvement in HbA1C and lipid profiles during ARA290 treatment as well as during the 56-day follow-up was observed. These data extend the findings in patients with sarcoidosis and indicate that in addition to a positive effect of IRR activation on neuropathic pain, the peptide improves the metabolic profile in patients with T2D (see also Ref. 42), which may have an additional positive effect on the affected small nerve fibers.

## 7. Conclusions

Selective activation of the IRR using engineered molecules offers the potential to decrease tissue damage while simultaneously augmenting repair over a wide range of disease processes. The small peptide ARA290, modeled from the 3-dimensional structure of EPO, has shown efficacy in preclinical models of disease, including neuropathy, without associated adverse effects. In these models, ARA290 has been shown to reprogram a proinflammatory, tissue-damaging milieu into one of healing and repair. The favorable effects of ARA290 administration to patients with neuropathic pain coupled with observed increases in small nerve fiber density, as assessed by examination of the cornea, are consistent with disease modification. However, to date, clinical observations have been limited to only 28 days of treatment. Only longer duration clinical trials with extended follow-up will provide the critical data with which to assess the full potential of IRR activation as a disease-modifying therapy.

## Conflict of interest statement

Leiden University received financial support from ARAIM Pharmaceuticals, Inc, to perform experimental and clinical studies on ARA290. M. Brines and A. Cerami are employees of ARAIM Pharmaceuticals, Inc, which is innate repair receptor agonists for clinical use. The other authors report no conflicts of interest to declare.
